# A plant-produced SARS-CoV-2 spike protein elicits heterologous immunity in hamsters

**DOI:** 10.3389/fpls.2023.1146234

**Published:** 2023-03-07

**Authors:** Emmanuel Margolin, Georgia Schäfer, Joel D. Allen, Sophette Gers, Jeremy Woodward, Andrew D. Sutherland, Melissa Blumenthal, Ann Meyers, Megan L. Shaw, Wolfgang Preiser, Richard Strasser, Max Crispin, Anna-Lise Williamson, Edward P. Rybicki, Ros Chapman

**Affiliations:** ^1^ Division of Medical Virology, Department of Pathology, Faculty of Health Sciences, University of Cape Town, Cape Town, South Africa; ^2^ Wellcome Trust Centre for Infectious Disease Research in Africa, University of Cape Town, Cape Town, South Africa; ^3^ Institute of Infectious Disease and Molecular Medicine, Faculty of Health Sciences, University of Cape Town, Cape Town, South Africa; ^4^ Biopharming Research Unit, Department of Molecular and Cell Biology, University of Cape Town, Cape Town, South Africa; ^5^ International Centre for Genetic Engineering and Biotechnology, Observatory, Cape Town, Cape Town, South Africa; ^6^ School of Biological Sciences and Institute of Life Sciences, University of Southampton, Southampton, United Kingdom; ^7^ Pathcare VetLab, Cape Town, South Africa; ^8^ Electron Microscope Unit, University of Cape Town, Cape Town, South Africa; ^9^ Division of Medical Virology, Faculty of Medicine and Health Sciences, Stellenbosch University Tygerberg Campus, Cape Town, South Africa; ^10^ Department of Medical Biosciences, University of the Western Cape, Cape Town, South Africa; ^11^ Department of Applied Genetics and Cell Biology, University of Natural Resources and Life Sciences, Vienna, Austria

**Keywords:** SARS-CoV-2, vaccine, glycoprotein, glycosylation, immunogenicity, challenge

## Abstract

Molecular farming of vaccines has been heralded as a cheap, safe and scalable production platform. In reality, however, differences in the plant biosynthetic machinery, compared to mammalian cells, can complicate the production of viral glycoproteins. Remodelling the secretory pathway presents an opportunity to support key post-translational modifications, and to tailor aspects of glycosylation and glycosylation-directed folding. In this study, we applied an integrated host and glyco-engineering approach, NXS/T Generation™, to produce a SARS-CoV-2 prefusion spike trimer in *Nicotiana benthamiana* as a model antigen from an emerging virus. The size exclusion-purified protein exhibited a characteristic prefusion structure when viewed by transmission electron microscopy, and this was indistinguishable from the equivalent mammalian cell-produced antigen. The plant-produced protein was decorated with under-processed oligomannose N-glycans and exhibited a site occupancy that was comparable to the equivalent protein produced in mammalian cell culture. Complex-type glycans were almost entirely absent from the plant-derived material, which contrasted against the predominantly mature, complex glycans that were observed on the mammalian cell culture-derived protein. The plant-derived antigen elicited neutralizing antibodies against both the matched Wuhan and heterologous Delta SARS-CoV-2 variants in immunized hamsters, although titres were lower than those induced by the comparator mammalian antigen. Animals vaccinated with the plant-derived antigen exhibited reduced viral loads following challenge, as well as significant protection from SARS-CoV-2 disease as evidenced by reduced lung pathology, lower viral loads and protection from weight loss. Nonetheless, animals immunized with the mammalian cell-culture-derived protein were better protected in this challenge model suggesting that more faithfully reproducing the native glycoprotein structure and associated glycosylation of the antigen may be desirable.

## 1 Introduction

The increasing incidence of viral outbreaks highlights the need for pandemic preparedness and the importance of investing in infrastructure development for vaccine manufacturing (Krammer, 2020). This is particularly relevant in low-income countries, such as those in Africa, where the capacity for end-to-end vaccine manufacturing is limited and where vaccines are almost exclusively sourced from wealthier countries (Margolin et al., 2020a). Accordingly, there is a clear need to establish sustainable and self-sufficient manufacturing sites in these vulnerable regions. However, in most cases the costs remain prohibitive – especially where manufacturing processes are reliant on mammalian cell culture systems which are especially expensive.

Molecular farming, the production of proteins in plants, has risen to prominence in recent years following efficacy reports of plant-made vaccines against influenza (Ward et al., 2020) and SARS-CoV-2 (Hager et al., 2022), and the therapeutic treatment of Ebola virus infection with plant-produced antibodies (Group et al., 2016). The use of plants as pharmaceutical bioreactors offers several advantages that lend themselves towards implementation in developing countries, including most notably lower infrastructure requirements(Fischer and Buyel, 2020) and potentially lower production costs (Rybicki, 2009; Murad et al., 2020). Furthermore, large-scale transient protein production in plants can be completed within weeks without the need to generate stable cell lines, which is time consuming and comparatively slower (D'Aoust et al., 2010). This presents an obvious advantage for responding to pandemic outbreaks where speed and scale are critical. Lastly, protein-based drugs typically require a less stringent cold-chain than other vaccine modalities, such as mRNA, which is an important consideration for resource-limited countries (Pambudi et al., 2022).

Given these advantages it is unsurprising that several plant-made vaccines against SARS-CoV-2 are at various stages of clinical development. The most advanced candidate is Medicago Inc.’s virus-like particle (VLP) vaccine which was approved for use in Canada in February 2022, and which demonstrated 69.5% efficacy against symptomatic disease and 78.8% efficacy against moderate-to-severe disease (Hager et al., 2022). These VLPs are comprised of a chimaeric spike where the native transmembrane and cytoplasmic tail regions have been replaced with the equivalent domains from influenza H5 haemagglutinin (Ward et al., 2021). This modification improves the formation of VLPs presenting the spike, which bud from the host cell without the need for any additional accessory proteins (Ward et al., 2021). Other noteworthy plant-derived candidates in clinical testing include a recombinant SARS-CoV-2 receptor-binding domain (RBD) antigen from Baiya Phytopharm (Phase 1, NCT05197712) and a RBD protein conjugated to a tobacco mosaic virus scaffold (Royal et al., 2021) from Kentucky BioProcessing, Inc. (Phase 1/2, NCT04473690). Similar success has also been described in preclinical studies from academic groups who have reported expression and immunogenicity of RBD antigens (Maharjan et al., 2021; Mamedov et al., 2021; Mardanova et al., 2021; Shin et al., 2021; Siriwattananon et al., 2021a; Siriwattananon et al., 2021b) and a full-length spike ectodomain that was produced by co-expression of human calreticulin (Margolin et al., 2022b). More recently, A SARS-CoV-2 VLP composed exclusively of the native spike has also been described (Jung et al., 2022).

Historically the production of complex glycoproteins in their native conformations, and particularly envelope viral glycoproteins, has posed a considerable challenge for molecular farming (Margolin et al., 2018), as plant-derived glycosylation is distinct from that of mammalian cells (Strasser et al., 2014; Strasser, 2016), and the glycoproteins from enveloped viruses typically have extensive disulfide bonding and a consequent significant dependence on host chaperones (Alonzi et al., 2017). In many cases viral envelope glycoproteins only accumulate at low levels in plants (Kang et al., 2018) (Margolin et al., 2018), and the recombinant products may be poorly folded and aberrantly glycosylated (Strasser et al., 2014) (Margolin et al., 2021a; Margolin et al., 2022a). These observations can largely be attributed to inadequacies in the plant glycosylation-directed folding pathways, which do not always adequately support the high levels of glycosylation (Margolin et al., 2021a), chaperone-mediated folding and processing (Wilbers et al., 2016; Margolin et al., 2020c; Margolin et al., 2022b) that are required by many viral glycoproteins. Furthermore, plant-produced glycoproteins often display unique features in their glycosylation including lower glycan occupancy compared to mammalian hosts, elevated oligomannose-type N-glycans, plant-specific complex N-glycans and unwanted N-glycan processing events (Strasser, 2016; Shin et al., 2017; Castilho et al., 2018; Margolin et al., 2021a).

In order to address these challenges and exploit the advantages inherent in plant-based protein production, there has been an increasing drive to develop novel host engineering approaches to support the folding and glycosylation requirements of complex viral glycoproteins (Margolin et al., 2020b; Margolin et al., 2020d). Constraints in the host chaperone machinery can be addressed by over-expression of chaperones to support critical folding events (Margolin et al., 2020c; Margolin et al., 2021b; Rosenberg et al., 2022), and recent evidence suggests that a combinatorial approach may confer additional benefit (Rosenberg et al., 2022). This approach typically results in increased glycoprotein accumulation and reduced ER stress, as the toxicity associated with the accumulation of misfolded protein is alleviated. Impaired proteolytic maturation can similarly be addressed by transient host engineering, and the co-expression of the protease furin has been shown to support efficient maturation of prototype viral glycoproteins which would not otherwise be properly cleaved in plants (Margolin et al., 2020c; Margolin et al., 2022b). The plant glycosylation machinery also imposes a bottleneck for the production of many viral glycoproteins, and aberrant glycosylation has been implicated in protein misfolding and aggregation (Margolin et al., 2021a; Margolin et al., 2022a). Foreign glyco-epitopes have been associated with hypersensitive reactions and some plant glycoproteins are allergens (Altmann, 2007; Arnold and Misbah, 2008); however, plant-specific glycosylation has been shown to be safe in volunteers immunized with plant-derived VLPs (Ward et al., 2014; Ward et al., 2020; Ward et al., 2021). These can similarly be addressed by remodelling the cellular machinery for glycosylation in the plant host – by the *in situ* provision of heterologous machinery where the plant cell glycosylation machinery is limiting and by the elimination of plant enzymes which impart expression system-dependent modifications (Margolin et al., 2020b; Margolin et al., 2020d) (Shin et al., 2017).

We previously developed a combinatorial host engineering platform, NXS/T Generation™, to produce well-folded viral glycoproteins in *N. benthamiana* with improved glycosylation (Margolin et al., 2022a). The expression technology revolves around the transient expression of the lectin binding chaperones calnexin or calreticulin (Protein Origami™) to support protein folding (Margolin et al., 2020c), which is combined with a series of glyco-engineering strategies to remodel the plant glycosylation machinery. These involve the co-expression of a single subunit oligosaccharyltransferase from *Leishmania major* (LmSTT3D) to enhance glycan occupancy (Castilho et al., 2018), an RNA interference construct to ablate truncation of glycans by an endogenous β-N-acetylhexosaminidase (Shin et al., 2017), and the use of a mutant strain of *N. benthamiana* ΔXF where the activity of α1,3-fucosyltransferase and β1,2-xylosyltransferase have been suppressed to prevent the formation of plant-specific complex glycans (Strasser et al., 2008). The resulting antigens are referred to as “glycan-enhanced” (GE) as they represent a notable improvement over the glycosylation of the protein when produced in the absence of this integrated host and glyco-engineering approach. Specifically, the GE proteins comprise of increased glycan occupancy and negligible undesired plant-specific modifications, which are associated with a concomitant improvement in protein structure, folding and oligomerization (Margolin et al., 2022a).

The NXS/T Generation^™^ platform was recently used to produce a soluble trimeric HIV envelope gp140 vaccine and the resulting GE antigen elicited largely equivalent immune responses in rabbits compared to the same antigen produced in mammalian cells (Margolin et al., 2022a). Encouraged by these observations, we initiated studies to investigate the broad applicability of this host-engineering platform to other pandemic and emerging viruses, including SARS-CoV-2. Given that direct comparisons between plant-produced and mammalian cell-produced viral glycoproteins are generally lacking, we sought to establish how closely the GE antigens resemble the equivalent protein when produced in mammalian cells, both in terms of glycosylation and immunogenicity. Previous work by our group and others has demonstrated that the co-expression of human calreticulin substantially improved the yields of a soluble SARS-CoV-2 spike ectodomain (Song et al., 2022; Margolin et al., 2022b), warranting integration of this approach with the glyco-engineering strategies that comprise the NXS/T Generation™ Platform. Therefore, in the present study we have built on this work by producing an improved spike antigen (HexaPro) using the NXS/T Generation™ platform. The resulting GE spike was then compared to the equivalent material produced in mammalian cells with regard to its structure and glycosylation. Finally, the vaccines were evaluated for their ability to elicit immunity against both the homologous SARS-CoV-2 wildtype (Wuhan) strain and the heterologous Delta variant, and to protect against viral challenge with the heterologous Delta variant (B.1.617.2).

## 2 Materials and methods

### 2.1 Gene design and expression constructs for protein production

A human-codon optimized variant of the HexaPro spike antigen was synthesized by GenScript using the sequence reported by Hsieh et al., which contains the following mutations F817P, A892P, A899P, A942, K986P & V987P and the furin cleavage site was replaced with a short linker sequence GSAS from amino acid 682 to 685 (PDB 6XKL). The antigen also contains a synthetic C-terminal foldon trimerization motif, an HRV3C protease recognition sequence, a twin Strep-tag and an octa-histidine sequence as reflected in the original manuscript (Hsieh et al., 2020). Synthetic HindIII and AgeI sites were incorporated at the 5’ end of the gene. EcoRI and XhoI sites were added to the 3’ end of the gene coding sequence. A Kozak sequence (CCACC) was incorporated into the sequence immediately upstream of the start codon of the gene. The gene was cloned into pMEx for mammalian cell expression (van Diepen et al., 2018) and pEAQ-*HT* for plant expression (Sainsbury et al., 2009) using HindIII and EcoRI or AgeI and XhoI, respectively. The recombinant pEAQ-*HT* plasmid was transformed into *A. tumefaciens* AGL1 by electroporation. Recombinant *A. tumefaciens* strains encoding LmSTT3D, human CRT and HEXO3RNAi have been described previously (Margolin et al., 2022a).

### 2.2 Production of SARS-CoV-2 HexaPro spike in plants

*N. benthamiana* ΔXF plants were propagated in flat trays at 25°C (55% humidity) under a controlled 16-hour light/8-hour dark photocycle, as described previously (Margolin et al., 2019). Recombinant spike was produced in *N. benthamiana* ΔXF by transient co-expression of the antigen with CRT, LmSTT3D and HEXO3RNAi, using *A. tumefaciens*-mediated infiltration to deliver the DNA coding sequences to the plant cells (Margolin et al., 2022a). The plant biomass was harvested 4 days post agroinfiltration and then homogenized in 2 buffer volumes of tris-buffered saline [pH 7.8], supplemented with 1% Depol 40 (Biocatalysts) and EDTA-free protease inhibitor (Roche). The resulting homogenate was incubated on an orbital shaker, at 4°C, for 1 hour and then filtered through Miracloth (Millipore Sigma) to remove insoluble plant debris. The pH was adjusted to 7.8 and the homogenate was clarified at 17000×g for 30 minutes. The sample was then filtered using a 0.45 µm Stericup-GP vacuum driven filter (Merck Millipore). The recombinant glycoprotein was captured using *Galanthus nivalis* lectin and trimeric spike was isolated by gel filtration, as described previously (Margolin et al., 2019).

### 2.3 Production of SARS-CoV-2 HexaPro in mammalian suspension cells

FreeStyle™ HEK293F cells (Invitrogen) were grown in sterile polycarbonate Erlenmeyer flasks on an orbital shaking platform set to 125 rpm. The cells were maintained at a density of 1-3×10^6^ cells/ml at 37°C, with 8% CO_2_. The cultures were passaged every 3-4 days, at a seeding density of 3×10^5^ cells/ml, using fresh FreeStyle™ 293 Expression Medium (Invitrogen). Spike protein was transiently expressed by transfecting cells, at a density of 1×10^6^ cells/ml, with 1 µg/ml of plasmid DNA. Polyethylenimine was used for transfections at a 3:1 ratio of transfection reagent:DNA. The culture media was harvested 5 days post-transfection and clarified by centrifugation at 2500×g, for 30 minutes. The clarified media was then filtered using a 0.45 µm Stericup-GP device (Merck Millipore). Spike trimers were purified as described for the plant-produced material.

### 2.4 Polyacrylamide gel electrophoresis and immunoblotting

Purified protein was resolved under denaturing conditions by SDS-PAGE as described previously (Margolin et al., 2019), and then immunoblotted using polyclonal mouse anti-His antibody (Serotech, MCA1396) at a 1:2000 dilution (Margolin et al., 2022b). Proteins were also resolved under native conditions using the BN-PAGE system followed by staining with BioSafe Coomassie G250 as previously reported (van Diepen et al., 2019).

### 2.5 Negative stain electron microscopy and image processing

Samples were pipetted onto glow-discharged (30 s in air) carbon-coated copper grids, washed three times in dH_2_O and stained with 2% uranyl acetate. For each sample ~30 images were collected using SerialEM at 2.2 Å/pixel using a Tecnai F20 transmission electron microscope fitted with a DE16 camera (Direct Electron, San Diego, CA USA) operated at 200 kV at an electron dose of ~50 e/Å^2^ and a defocus of −1.5 μm. Relion 3.1 (Scheres, 2012) was used for image processing and 3D reconstruction: briefly ~1 000 particles were manually picked and used to generate 2D class averages for reference-based picking yielding ~4000 particles. This particle set was refined using 2D classification and used to generate a *de novo* initial model using stochastic gradient descent (Punjani et al. 2017). Following refinement (final resolution: ~20 Å), UCSF Chimera (Pettersen et al., 2004) was used for three-dimensional visualization and rendering.

### 2.6 Site-specific N-glycan analysis of purified spike

Aliquots of spike protein were denatured for 1 h in 50 mM Tris/HCl, pH 8.0 containing 6 M of urea and 5 mM dithiothreitol (DTT). Next, spike proteins were alkylated by adding 20 mM iodoacetamide (IAA) and incubated for 1 h in the dark, followed by a 1 h incubation with 20 mM DTT to eliminate residual IAA. The alkylated spike proteins were buffer exchanged into 50 mM Tris/HCl, pH 8.0 using Vivaspin columns (3 kDa) and digested separately overnight using trypsin, chymotrypsin (Mass Spectrometry Grade, Promega), or alpha lytic protease (Sigma Aldrich) at a ratio of 1:30 (w/w). The next day, the peptides were dried and extracted using C18 Zip-tip (MerckMillipore). The peptides were dried again, resuspended in 0.1% formic acid and analyzed by nanoLC-ESI MS with an Ultimate 3000 HPLC (Thermo Fisher Scientific) system coupled to an Orbitrap Eclipse mass spectrometer (Thermo Fisher Scientific) using stepped higher energy collision-induced dissociation (HCD) fragmentation. Peptides were separated using an EasySpray PepMap RSLC C18 column (75 µm × 75 cm). A trapping column (PepMap 100 C18 3 μM, 75 μM × 2 cm) was used in line with the liquid chromatography (LC) before separation with the analytical column. The LC conditions were as follows: 275 min linear gradient consisting of 0%–32% acetonitrile in 0.1% formic acid over 240 min followed by 35 minutes of 80% acetonitrile in 0.1% formic acid. The flow rate was set to 300 nl/min. The spray voltage was set to 2.5 kV and the temperature of the heated capillary was set to 40°C. The ion transfer tube temperature was set to 275°C. The scan range was 375 − 1500 m/z. Stepped HCD collision energy was set to 15%, 25%, and 45%, and the MS2 for each energy was combined. Precursor and fragment detection were performed using an Orbitrap at a resolution MS1= 120,000, MS2 = 30,000. The AGC target for MS1 was set to standard and injection time set to auto, which involves the system setting the two parameters to maximize sensitivity while maintaining cycle time. Full LC and mass spectrometry (MS) methodology can be extracted from the appropriate Raw file using XCalibur FreeStyle software or upon request.

Glycopeptide fragmentation data were extracted from the raw file using Byos (Version 3.5; Protein Metrics Inc.). The glycopeptide fragmentation data were evaluated manually for each glycopeptide; the peptide was scored as true-positive when the correct b and y fragment ions were observed along with oxonium ions corresponding to the glycan identified. The MS data was searched using the Protein Metrics 309 N-glycan library with sulfated glycans added manually combined with the Protein metrics 57 Plant N-linked glycan library. The relative amounts of each glycan at each site, as well as the unoccupied proportion were determined by comparing the extracted chromatographic areas for different glycotypes with an identical peptide sequence. All charge states for a single glycopeptide were summed. The precursor mass tolerance was set at 4 and 10 p.p.m. for fragments. A 1% false discovery rate was applied. The relative amounts of each glycan at each site as well as the unoccupied proportion were determined by comparing the extracted ion chromatographic areas for different glycopeptides with an identical peptide sequence. Glycans were categorized according to the composition detected. Chimera X v1.3 was used to visualize and represent the glycosylation of the S protein (Pettersen et al., 2021), using the .pdb file produced by (Zuzic et al., 2022), using S_full_domgly as the template.

HexNAc(2)Hex(10+) was defined as M9Glc, HexNAc(2)Hex(9–4) was classified as oligomannose-type. Any of these structures containing a fucose were categorized as FM (fucosylated mannose). HexNAc(3)Hex(5-6)X was classified as Hybrid with HexNAc(3)Hex(5-6)Fuc(1)X classified as Fhybrid. Complex-type N-glycans were classified according to the number of HexNAc subunits and the presence or absence of fucosylation. As this fragmentation method did not provide linkage information compositional isomers were grouped, so for example a triantennary glycan contained HexNAc 5 but so did a biantennary glycan with a bisect. Core glycans refer to truncated structures smaller than M3. Any compositions containing a monosaccharide corresponding to a pentose (e.g., Xylose) were classified in the pentose category. Likewise, any glycan composition detected containing at least one fucose or sialic acid were assigned as “Fucose” and “NeuAc,” respectively.

### 2.7 Isolation, propagation and titration of SARS-CoV-2, delta variant

All work involving live SARS-CoV-2 was performed inside an accredited Biosafety Level 3 facility in accordance with the safety regulations regarding risk level 3 pathogens (WHO, 2021). SARS-CoV-2-positive patient samples were obtained from the National Health Laboratory Service (NHLS), Tygerberg, Cape Town, South Africa, and the lineage was confirmed to be SARS-CoV-2 Delta at Stellenbosch University (SU) as part of the Network for Genomic Surveillance in South Africa (NGS-SA) initiative (Msomi et al., 2020).

Vero E6 cells were maintained in Dulbecco’s modified Eagle medium (DMEM) containing sodium pyruvate and L-glutamine (PAN Biotech, Aidenbach, Germany) with 10% foetal bovine serum (Gibco, Texas, USA) and 1% each of non-essential amino acids (Lonza, Basel, Switzerland), amphotericin B (Gibco, Texas, USA) and penicillin/streptomycin (PAN Biotech, Aidenbach, Germany). Vero E6 cells were grown at 37°C and 5% CO_2_ and were passaged every 3-4 days.

For virus isolation, Vero E6 cells were seeded at 3.5x10^5^ cells/ml in a 6-well plate 18-24 hrs before infection. After one wash with 1xDPBS (PAN Biotech, Aidenbach, Germany) the cells were inoculated with patient sample that was diluted 1:5 in DMEM. The inoculum was removed after one hour incubation at room temperature, and the cells were washed once with 1xDPBS before the addition of post-infection media (DMEM, 2%FBS, 1% each of non-essential amino acids, amphotericin B, penicillin/streptomycin). The cells were incubated at 37°C with 5% CO_2_ and monitored daily for 3-7 days or until >90% cytopathic effect (CPE) was observed. The cell culture supernatant was then harvested and used to infect freshly seeded Vero E6 cells to produce a second passage stock of the virus, and then a third passage stock. The third passage stock was sequence confirmed at SU using Oxford Nanopore Technology, as described previously (Maponga et al., 2022), to ensure no mutations had been introduced during passaging of the virus. The viral RNA load was quantified using a quantitative real time PCR assay specific for the E gene, as described by Corman et al. (Corman et al., 2020), and the infectious virus titer was determined using a standard plaque assay on Vero E6 cells.

### 2.8 Hamster vaccination and intranasal infection

Immunization and challenge experiments were carried out using 6–9-week-old male or female Syrian Golden Hamsters (*Mesocricetus auratus)*. All experimental procedures were conducted in the Research Animal Facility at the University of Cape Town, in accordance with AEC 021_005. Animal immunizations took place under BSL-2 conditions, whereas viral challenge experiments were confined to the BSL-3 laboratory using the IsoRAT900 Biocontainment system. Prior to challenge experiments, the Delta variant of SARS-CoV-2 was tested to determine the optimal inoculum for infection. Groups of 5 hamsters were inoculated intranasally with 10^4^ PFU or 10^5^ PFU virus or an equivalent volume of PBS. The body weights of the animals were monitored daily, and oropharyngeal swabs were collected prior to challenge and on days 3 and 6 post infection. The experiment was terminated on day 14 post-infection and organs were harvested for histological investigation.

The vaccine challenge study was conducted using 5 hamsters per experimental group. Animals were immunized intramuscularly with 5 µg of protein, formulated 1:1 in Alhydrogel^®^ adjuvant (van Diepen et al., 2018; Margolin et al., 2022b), on days 0 and 28. The experimental control group was immunized with an equivalent volume of PBS. Blood was drawn on day 0, 14 and 46. Vaccinated animals were transferred to the IsoRAT900 biocontainment system in the BSL3 laboratory on day 46 and were intranasally infected with 10^4^ PFU of the Delta variant of SARS-CoV-2 on day 49. Oropharyngeal swabs were collected on days 46 (prior to infection), 52 (3 days post infection) and 55 (5 days post infection). The weight of the hamsters was recorded prior to both vaccination and infection, and then monitored daily following infection, until the experimental endpoint. The experiment was terminated on day 55. Lungs were collected in 10% buffered formalin.

### 2.9 Neutralization assay

Neutralizing antibodies against wild type/Wuhan and Delta viruses were quantified at weeks 0, 14 and 46 using a pseudovirus neutralization assay, as described previously (Margolin et al., 2022b). SARS-CoV-2 pseudovirions were generated in HEK-293T cells by co-transfection of plasmids pNL4-3.Luc.R-.E- (aidsreagent #3418) and pcDNA3.3-SARS-CoV-2-spike Δ18 (Wuhan strain) (Rogers et al., 2020) or pcDNA3.3-SARS-CoV-2-spike Δ18 – Delta (Delta variant), respectively. The latter was derived from plasmid pcDNA3.3-SARS-CoV-2-spike Δ18 (Wuhan strain) by side-directed mutagenesis using the QuikChange Lightning Multi Site-Directed Mutagenesis Kit (Agilent) together with the primers T19R (agccagtgtgtgaacctgaggaccaggaccc), R158G (aacaagtcctggatggagtctggggtctactcc), L452R (aggcaactacaactaccgctacagactgttcagga), T478K (atttaccaggctggcagcaaaccatgtaatggagt), D614G (gctgtgctctaccagggtgtgaactgtactgag), P681R (acccagaccaacagcagaaggagggcaaggtc), D950N (ccctgggcaaactccaaaatgtggtgaaccagaa), 156_157_del_F (acaacaagtcctggatggagtctagggtctactcc), and 156_157_del_R (ggagtagaccctagactccatccaggacttgttgt). Neutralization titres were reflected as the half maximal inhibitory dilution for each plasma sample.

### 2.10 Determination of SARS-CoV-2 viral loads

SARS-CoV-2 viral loads were determined from nasal swabs sampled in 500µl QVL Lysis Buffer containing 10µg/ml carrier RNA (Poly A) (Omega Bio-Tek). MS2 Phage Control (TaqMan™ 2019-nCoV Control Kit v2 (Applied Biosystems)) was added to each sample prior to RNA extraction as an internal positive control. RNA isolation was performed using the E.Z.N.A.^®^ Viral RNA Kit (Omega Bio-Tek). This was followed by multiplex qRT-PCR of the isolated RNA on a QuantStudio™ 7 Flex Real-Time PCR System (Thermo Fisher) using the TaqMan™ 2019-nCoV Control Kit v2 (Applied Biosystems) including serial dilutions of known SARS-CoV-2 viral RNA ranging from 1x10^4^ copies/µl to 1x100 copies/µl. Viral loads were calculated based on the standard curve and expressed as copies/µl.

### 2.11 Histopathology

The lungs were sectioned and stained routinely with H&E for histopathology. Tissue was evaluated by light microscopy for evidence of necrosis/inflammation and/or repair/fibrosis. Lesions in the lung were graded as either absent (0), minimal (1), mild (2), moderate (3), marked (4) or severe (5). The severity of histopathology was graded based on observations of 1) lympho-plasmacytic infiltration, 2) bronchiolitis/peribronchiolitis, alveolitis and bullous emphysema, 3) vasculitis/perivasculitis and 4) atelectasis.

### 2.12 Statistical analyses

All statistical analyses were conducted using GraphPad Prism 9. Statistical comparisons between groups over time were analysed using a two-way Anova test whereas statistical comparisons between two groups at a single time point were made using a multiple unpaired t-test. A p value of <0.05 was considered as the threshold of significance for all statistical tests. The half maximal inhibitor dilution (ID_50_) of sera samples was determined using a non-linear regression.

## 3 Results

### 3.1 Production of a glycan-enhanced SARS-CoV-2 spike in plants

The SARS-CoV-2 spike protein is a typical trimeric class 1 viral fusion protein which is initially synthesised as a single chain precursor of ~1300 amino acids that is cleaved by host proteases, such as furin, into two subunits, S1 and S2 (Duan et al., 2020; Zhang et al., 2021; Yan et al., 2022). The cleavage is essential for cell fusion and viral infectivity (Yan et al., 2022). The mature glycoprotein, on the viral surface, binds to the angiotensin-converting enzyme 2 (ACE2) receptor on the host cell membrane. This complex is then cleaved by the type 2 transmembrane protease TMPRSS2, triggering rearrangement of the protein from a metastable prefusion conformation to a stable postfusion conformation, activating the spike protein and enabling entry into the host cell (Duan et al., 2020; Zhang et al., 2021; Yan et al., 2022).

**Figure 1:**
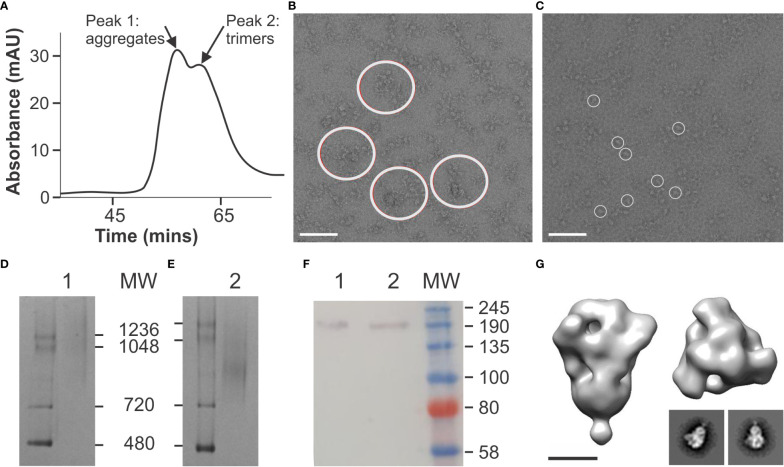
Production of a soluble, stabilized spike in plants. **(A)** Gel filtration profile of affinity-captured spike. Peak 1, fractions 31-35 = aggregates; Peak 2, fractions 36-40 = trimers. **(B)** Negative stain electron microscopy of fractions 31-35 derived from peak 1 (aggregates) in A. Large circles indicate large, clumps of aggregated spike protein. **(C)** Negative stain electron microscopy of fractions 36-40 derived from peak 2 (trimers) in A. Small circles indicate spike trimers. **(D)** Coomassie-stained BN-PAGE of fractions 31-35, peak 1 (aggregates) in A. **(E)** Coomassie-stained BN-PAGE of fractions 36-40, peak 2 (trimers) from A. **(F)** Western blotting of fractions 31-35 (lane 1) and 36-40 (lane 2) from peaks 1 and 2 respectively in A using polyclonal mouse anti-His antibody [Serotech, MCA1396]. **(G)** Three-dimensional reconstruction and class averages of purified trimeric spike protein (fractions 36-40) from electron micrographs shown in **(C)**.

In order to produce the SARS-CoV-2 spike antigen in plants, we implemented an integrated suite of approaches that were previously developed to transiently remodel the plant secretory pathway to address host constraints in glycosylation and glycosylation-directed folding (NXS/T Generation™) (Margolin et al., 2022a). We applied this platform to produce a spike construct (HexaPro) that had been engineered to stabilize the antigen in the prefusion conformation by the incorporation of 6 proline mutations (Hsieh et al., 2020). Following affinity-chromatography and gel filtration (**Figure 1A**), aggregated material (fractions 31-35) was discarded (**Figure 1B**) and a homogenous population of well-ordered trimers (fractions 36-40) was recovered (**Figure 1C**). Coomassie-stained BN-PAGE gels verified the gel filtration peaks comprised of aggregates and trimers respectively (**Figures 1D, E**) and Western blotting confirmed the identity of the purified material with expected products observed at ~180 kDa (**Figure 1F**). Two-dimensional class averages and three-dimensional reconstruction of the purified spike trimers yielded a typical “kite-like” structure, which projected from a narrow base that would be proximal to the membrane in the context of the native virus (**Figure 1G**). When viewed from above the reconstruction depicted characteristic 3-fold symmetry and was consistent with published accounts for the antigen (Hsieh et al., 2020). The HexaPro spike was also produced in FreeStyle HEK293F mammalian cells as a control. The protein yielded equivalent structures that were consistent with previously published accounts (Hsieh et al., 2020), and which were indistinguishable from the plant-derived spike (**Figure S1**).

### 3.2 Site-specific glycan analysis of spike produced using the NXS/T generation™ system

**Figure 2:**
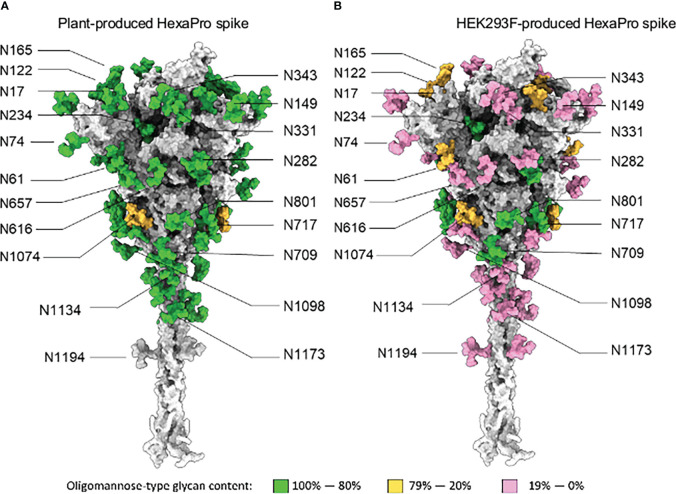
Site-specific N-glycosylation of plant and HEK293F-produced spike. **(A)** Fully glycosylated model of the SARS-CoV-2 spike glycoprotein in a native-like prefusion conformation, generated previously by (Zuzic et al., 2022). The individual N-glycan sites were colored according to the site-specific abundance of oligomannose-type glycans determined by LC-MS for spike protein produced using glycan enhanced *N.benthamiana* cells. **(B)** Model of the SARS-CoV-2 S protein produced in an identical manner to panel A except colored according to LC-MS analysis of spike protein produced by transient transfection of HEK293F cells.

**Figure 3:**
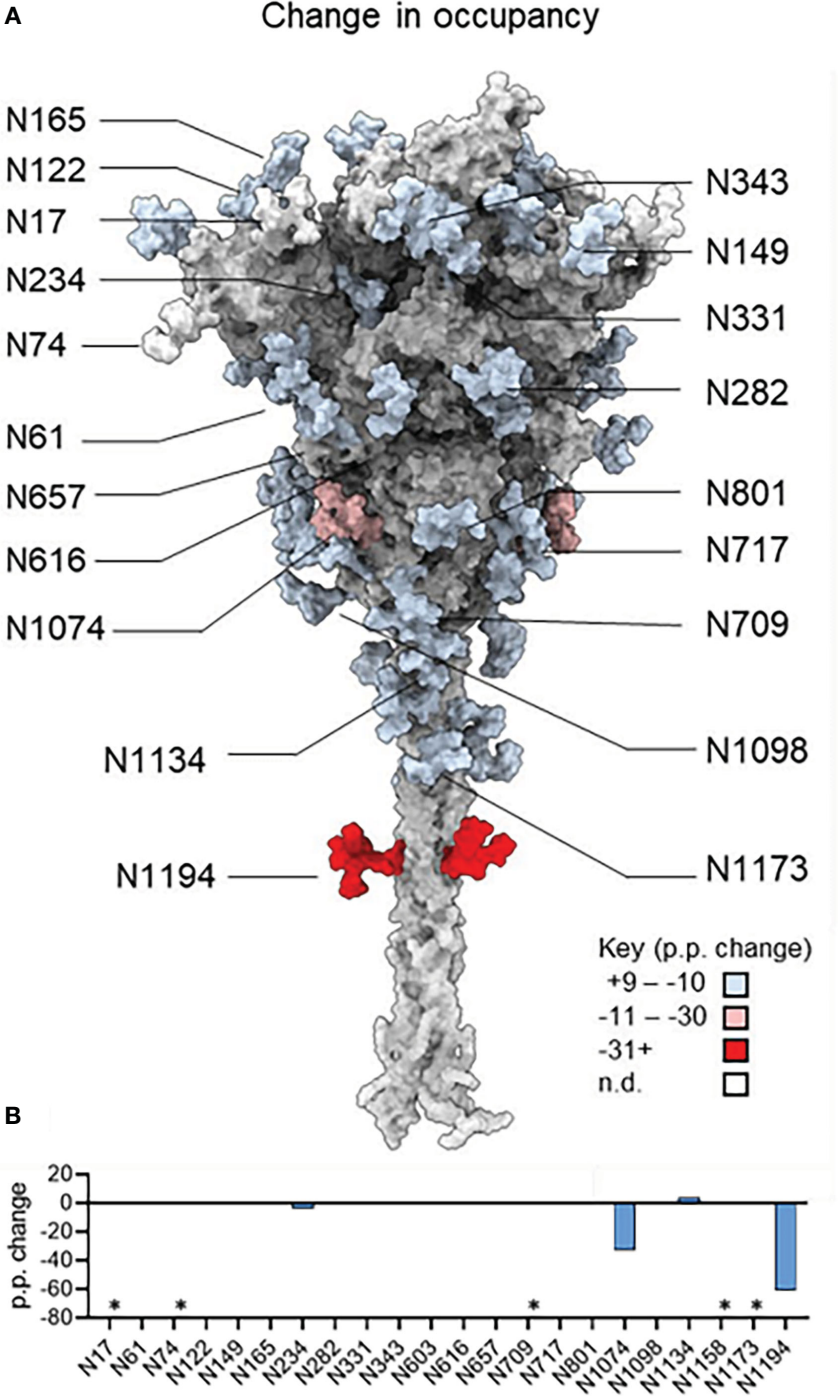
The change in site-specific N-glycan occupancy of plant-produced and mammalian cell-derived spike. **(A)** Model of the SARS-CoV-2 S glycoprotein in the native-like prefusion conformation generated by Zuzic et al., (2022). Individual N-glycan sites are colored according to the percentage point change in glycan occupancy of plant-derived protein relative to the comparator HEK293F-produced protein and therefore positive and negative values represent a relative increase or decrease in the glycan occupancy each sequon. **(B)** Bar graph depicting the percentage point changes depicted in panel A. A negative value indicates lower glycan occupancy in the plant-produced spike protein. Sites that could not be resolved are denoted with an asterisk.

The site-specific N-glycosylation of the plant-produced antigen was determined using a previously developed analytical pipeline (**Figures 2A**, **S2** and **Table S1**) (Margolin et al., 2021a). The protein was decorated almost exclusively with oligomannose-type N-glycans, which exhibited low levels of glycan processing. The N-glycosylation of the control mammalian protein was similarly determined (**Figures 2B**, **S3** and **Table S2**). The mammalian cell-produced spike contained large amounts of complex N-glycans, as well as incompletely processed oligomannose glycans at several N-glycosylation sites (N61, N122, N165, N234, N603, N616, N709, N717, N801, N1074). Minor populations of core glycans were also observed at 3/22 sites in the plant-produced protein (N61, N331 and N616) which were absent when the protein was produced in HEK293F cells. These structures comprise of Hex(3)HexNAc(2) or smaller structures which arise from enzymatic cleavage of the glycan. The N-glycan site occupancy between the 2 systems was largely comparable (**Figure 3** and **Table S3**). Partial unoccupied sites were observed at N234 (4%), N1074 (34%), N1098 (1%) and N1194 (61%) in the plant-produced protein (**Table S1**).

### 3.3 Immunogenicity of plant-produced spike in hamsters

**Figure 4:**
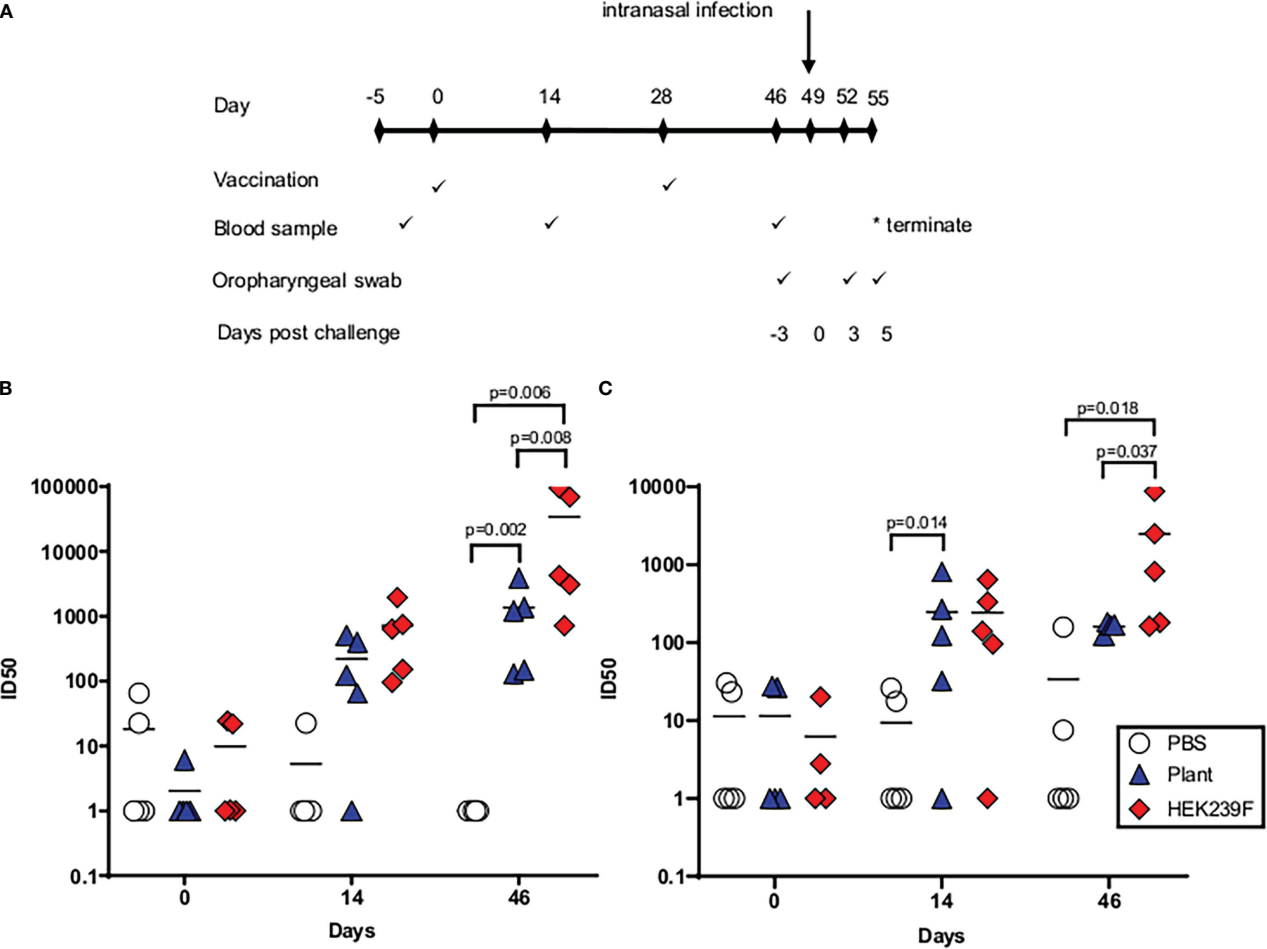
Immunogenicity of spike trimers in hamsters when produced in plants and mammalian cells. **(A)** Immunization schematic depicting the timing of immunizations and sampling during the experiment. **(B)** Neutralizing antibody titres (ID_50_) against the matched wildtype (Wuhan) viral isolate. **(C)** Neutralizing antibody titres (ID_50_) against heterologous Delta virus. Pre-bleed= day -2, first bleed = day 14, final bleed = day 46.

Hamsters were immunized with 5 µg of purified trimer, formulated in Alhydrogel^®^, to compare the immunogenicity of the vaccine when produced in plants and mammalian cells (**Figure 4A**). The vaccines both elicited neutralizing antibodies against the matched wild type virus, and against the heterologous Delta virus in all immunized animals after the second injection (**Figures 4B, C**, respectively). With the exception of a single animal in the plant group, all hamsters developed detectable neutralizing antibodies against the Wuhan virus after a single immunization (**Figure 4B**). Similarly, 4/5 animals in each group developed neutralizing antibodies against the Delta virus after a single immunization (**Figure 4C**). The mammalian cell-derived protein induced significantly higher mean titers of neutralizing antibodies against both viral isolates when compared to the plant protein. Irrespective of the producing system, heterologous neutralizing antibody titres were lower than the titres observed against the vaccine-matched virus (**Figure 4C**).

**Figure 5:**
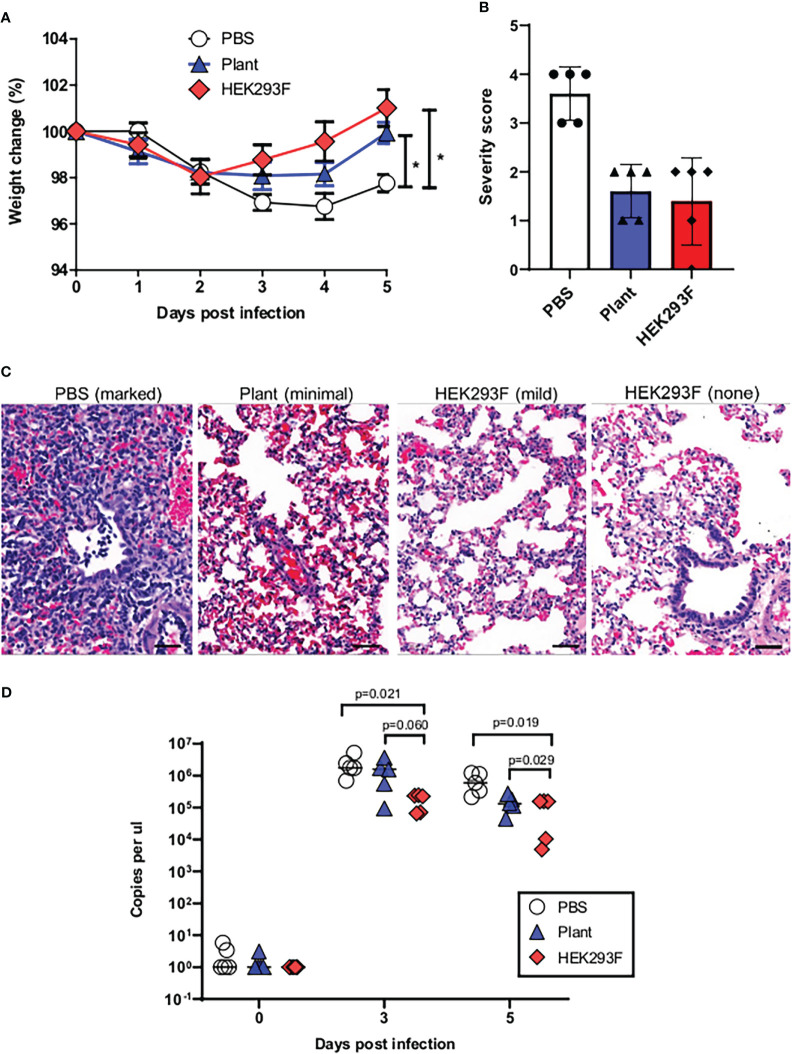
Vaccine-mediated protection against heterologous challenge. **(A)** Change in weight following experimental challenge. **(B)** Grading of lung pathology following challenge. **(C)** Representative images of histopathology findings in lung sections. **(D)** Viral load following challenge.

The hamsters were then challenged with the heterologous Delta virus to emulate the world scenario at the time of the experiment where the approved vaccines were based on the original wild type virus and the predominating variant was Delta. Following challenge, a slight decrease in body weight was witnessed in all groups over the first 2 days with no appreciable differences observed between vaccinated and unvaccinated animals. Both vaccinated groups rapidly recovered the lost weight, whereas the weights of the unvaccinated hamsters continued to decline until the experimental endpoint when a slight increase was observed. Both vaccines afforded significant protection against weight loss compared to the control group which demonstrated protracted weight loss and slower recovery (p<0.05) (**Figure 5A**). Animals immunized with the mammalian cell-produced antigen demonstrated a trend towards faster recovery of the lost weight, although this was not statistically significant (p>0.05).

Pathology of lung tissue following challenge was graded based on the observed severity which was reflected as a numerical score of 0-5 where 0 indicates that no significant histological changes were observed and 5 indicates severe changes (**Figures 5B**, **S4**). Moderate to marked microscopic lesions were observed in all unvaccinated animals. In contrast, the majority of animals from both vaccinated groups showed only mild or minimal changes on histopathology (**Figure 5C**). A single animal that was vaccinated with the mammalian cell-produced protein demonstrated complete protection from any observable changes on histopathology. Both vaccines resulted in lower viral loads following challenge when compared to the control group, and this was observed at both time points when viral load was assayed (**Figure 5D**). Animals vaccinated with the mammalian cell produced spike had a trend towards lower viral loads than animals immunized with the plant-produced spike, although this was only significant at the final time point.

## 4 Discussion

Remodelling the host cell factory has shown promise as a novel paradigm to produce increasingly complex biopharmaceuticals in plants (Frigerio et al., 2022; Ruocco and Strasser, 2022; Ganesan et al., 2023). These approaches enable intrinsic constraints in the host machinery to be addressed resulting in increased accumulation, enhanced folding, improved glycosylation and even tailor-made post-translational modifications (Margolin et al., 2020d). This has important implications for vaccine development and accordingly plant host engineering has shown promise in the production of novel vaccine antigens (Margolin et al., 2020c; Rosenberg et al., 2022; Song et al., 2022; Margolin et al., 2022a; Margolin et al., 2022b; Ruocco and Strasser, 2022; Margolin et al., 2020d) including those from enveloped viruses which historically have posed a challenge for plant-based production.

Following a series of studies to identify constraints in glycosylation-directed folding pathways in plants (Margolin et al., 2020c; Margolin et al., 2021a; Margolin et al., 2022b), we previously developed an integrated approach, NXS/T Generation™, to support improved viral glycoprotein production in *N. benthamiana*. In a proof-of-concept study, we recently used this technology platform to produce a soluble HIV envelope gp140 trimer, which elicited comparable immune responses to the cognate mammalian protein in a rabbit immunogenicity model (Margolin et al., 2022a). In the present study, we applied this approach to the prototype spike glycoprotein from SARS-CoV-2 and compared the resulting protein to the antigen when it was produced in mammalian cells. This work serves as part of a larger initiative to evaluate the broader applicability of the NXS/T Generation™ technology.

Following production in plants using this platform, the proteins observed by NSTEM were consistent with the prefusion trimer of the SARS-CoV-2 spike (Hsieh et al., 2020), and these were indistinguishable with what was observed for the mammalian control protein. However, despite the apparent similarities, juxtaposition of the site-specific N-glycosylation of the two proteins revealed substantial differences. Whilst the plant-derived protein contained an abundance of poorly processed oligomannose structures, the mammalian cell-derived control displayed a high degree of N-glycan processing with an abundance of mature complex glycans. Other studies have also reported reduced mannose processing in plants for viral glycoproteins (Dobrica et al., 2021; Margolin et al., 2021a), although the extent to which processing was arrested here remains a surprise. Interestingly the N-glycosylation pattern observed for the plant-produced spike in this study contrasts with a recent report describing the production of virus-like particles bearing the full-length protein in wild type *N. benthamiana* (Balieu et al., 2022). In the study conducted by Balieu et al., highly processed mature N-glycans were observed, including typical plant-derived complex glycans with Lewis A epitopes, contrasting against the abundance of immature oligomannose N-glycans that we observed. Although the use of *N. benthamiana* ΔXF as an expression host in the present study would preclude the formation of typical plant complex N-glycans carrying β1,2-xylose and core α1,3-fucose, it should not undermine normal glycan processing along the secretory pathway, and therefore the predominance of unprocessed oligomannose-type N-glycans is puzzling. It is worth noting that the spike protein in this study was soluble and therefore would not have the same association with the endomembrane system as would be expected for a full-length antigen. In the case of the HIV Env glycoprotein, membrane-bound Env has been reported to exhibit enhanced glycan processing and maturation compared to the cognate soluble protein, and this is thought to result from improved association with the membrane-bound host processing machinery (Allen, 2021). Although this could be a contributory factor, it seems unlikely to account for the dramatic retardation of glycan maturation that we observed. Although decreased glycan processing was also observed in our previous work with HIV Env using this production platform, this was less apparent as the extensive glycosylation of the glycoprotein sterically hinders glycan-processing leading to an already elevated oligomannose content (Pritchard et al., 2015; Margolin et al., 2022a).

More encouragingly, the site-specific glycan occupancy of the plant-derived material was similar to that of the mammalian protein and the extensive under glycosylation that we previously observed for viral glycoproteins produced without the NXS/T Generation™ platform (Margolin et al., 2021a) was not seen here. This can be attributed to the co-expression of *Leishmania* LmSTT3D which has been demonstrated to improve glycan occupancy of a broad range of substrates in plants (Castilho et al., 2018; Margolin et al., 2022a).

The impact of impaired glycan maturation on vaccine immunogenicity is largely speculative and probably depends on the antigen in question. In the previous study with HIV, elevated high-mannose glycans did not appear to negatively impact the immunogenicity of the plant-produced vaccine – although it is acknowledged that the sample size was small (Margolin et al., 2022a) and that the antigen naturally displays increased mannose content (Pritchard et al., 2015). Accordingly, it is less clear how this would impact immunogenicity in the context of an antigen, which would typically have complex glycans as the predominating species, such as the SARS-CoV-2 spike (Chawla et al., 2022). However, robust immune responses have been generated to SARS-CoV-2 spike proteins derived from a wide range of different sources with different glycosylation profiles (Chawla et al., 2022).

Although the plant-produced antigen was immunogenic, hamsters immunized with the vaccine developed significantly lower titres of neutralizing antibodies than animals that were immunized with the mammalian cell-produced protein. This is not unprecedented and these observations mirror studies with influenza virus where haemagglutinin antigens containing high-mannose glycans elicited lower haemagglutinin inhibition antibody titres than equivalent antigens containing typical mammalian-type complex glycans (de Vries et al., 2012). Nonetheless, the plant-produced vaccine still elicited cross-neutralizing antibodies against the Delta variant of SARS-CoV-2.

Following heterologous challenge, both plant-produced and mammalian cell-derived vaccines conferred significant protection from disease. The initial loss of weight indicates that the hamsters were not protected from infection but were able to clear the virus, probably *via* a T cell response. This protected against lung pathology. We had previously reported strong T cell responses to this plant made vaccine in mice (Margolin et al., 2021b). However, the mammalian-cell produced antigen elicited superior protection across multiple parameters including weight change, viral load and lung pathology. This can potentially be attributed to the observed differences in glycosylation. It has been shown that exposure of mannose residues can promote protein turnover, which would potentially result in reduced half-life of the plant-produced protein (Yang et al., 2015). This would in turn result in reduced antigenic stimulation compared to the mammalian cell-derived product, which contains significantly reduced mannose by comparison. In the context of HIV Env, it has also been shown that the abundance of mannose residues impairs immune responses from dendritic cells (Shan et al., 2007) and that enzymatic elimination of these mannose structures can increase the immunogenicity of an Env-derived gp120 antigen (Banerjee et al., 2009). It is unclear if a similar phenomenon is occurring here and further work is required to delineate the mechanism by which the glycosylation impairs the immune response. Lastly, although the differences in immunogenicity have been attributed to the differential glycosylation patterns from the expression hosts, we cannot discount expression-system dependent differences in folding which would also influence vaccine immunogenicity. We also note the limited resolution afforded by negative stain electron microscopy, and cryo-electron microscopy would be required to determine whether meaningful structural differences exist. In conclusion, this study highlights the utility of the NXS/T generation™ platform for remodelling the plant secretory pathway to produce complex viral glycoproteins in plants. Nonetheless, further refinement of the technology could greatly enhance vaccine immunogenicity and this could potentially even be used to generate vaccines with tailor-made glycosylation. Finally, this work highlights the influence of glycosylation in vaccine immunogenicity and reinforces this as an important consideration for production of important vaccine antigens by molecular farming.

## Data availability statement

The original contributions presented in the study are included in the article/**Supplementary Material**. Further inquiries can be directed to the corresponding authors.

## Ethics statement

The animal study was reviewed and approved by Animal Ethics Committee of the University of Cape Town, South Africa.

## Author contributions

EM conceptualized the study with input from A-LW, RC and ER. Protein production was conducted by EM. JW carried out the negative stain electron microscopy and image processing. JA completed the site-specific N-glycan analysis. AS propagated and titrated the SARS-CoV-2 virus, under the supervision of MS and WP. RC managed the hamster experiment. Immunogenicity assays were conducted by EM and GS. MB cloned the Delta variant of the Spike protein for production of the pseudovirions used for neutralization assays. GS determined the viral loads and performed the neutralization assays. SG carried out the histopathology. EM drafted the manuscript. All authors contributed to data analysis and reviewed the final manuscript before submission. Funding for the project was obtained by RC, EM, MS, WP and A-LW. All authors contributed to the article and approved the submitted version.
