# Supplementary material for: A plant-produced SARS-CoV-2 spike protein elicits heterologous immunity in hamsters
Source: Front Plant Sci. 2023 Mar 7;14:1146234. doi: 10.3389/fpls.2023.1146234 (PMC10028082; doi:10.3389/fpls.2023.1146234)
Supplement: Supplementary file 1 [file DataSheet_1.pdf]

Supplementary Material

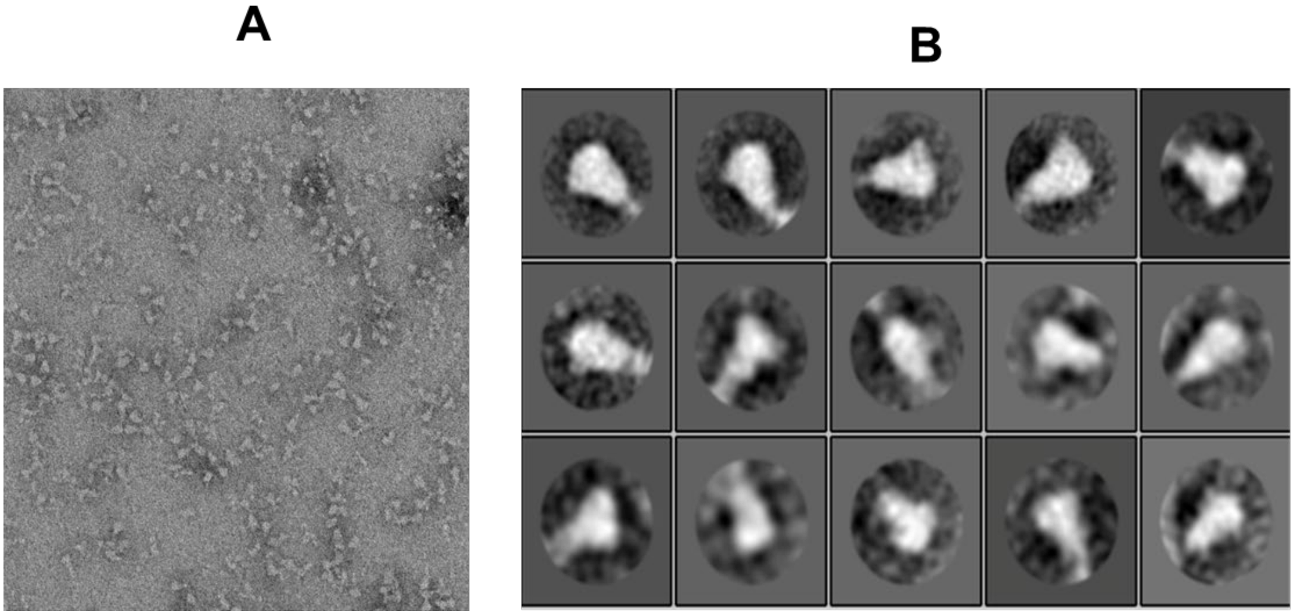

Supplementary Figure S 1. Negative stain electron microscopy images of purified spike trimers produced by transient transfection of HEK293-F cells. A) Raw micrograph, B) 2D class averages.

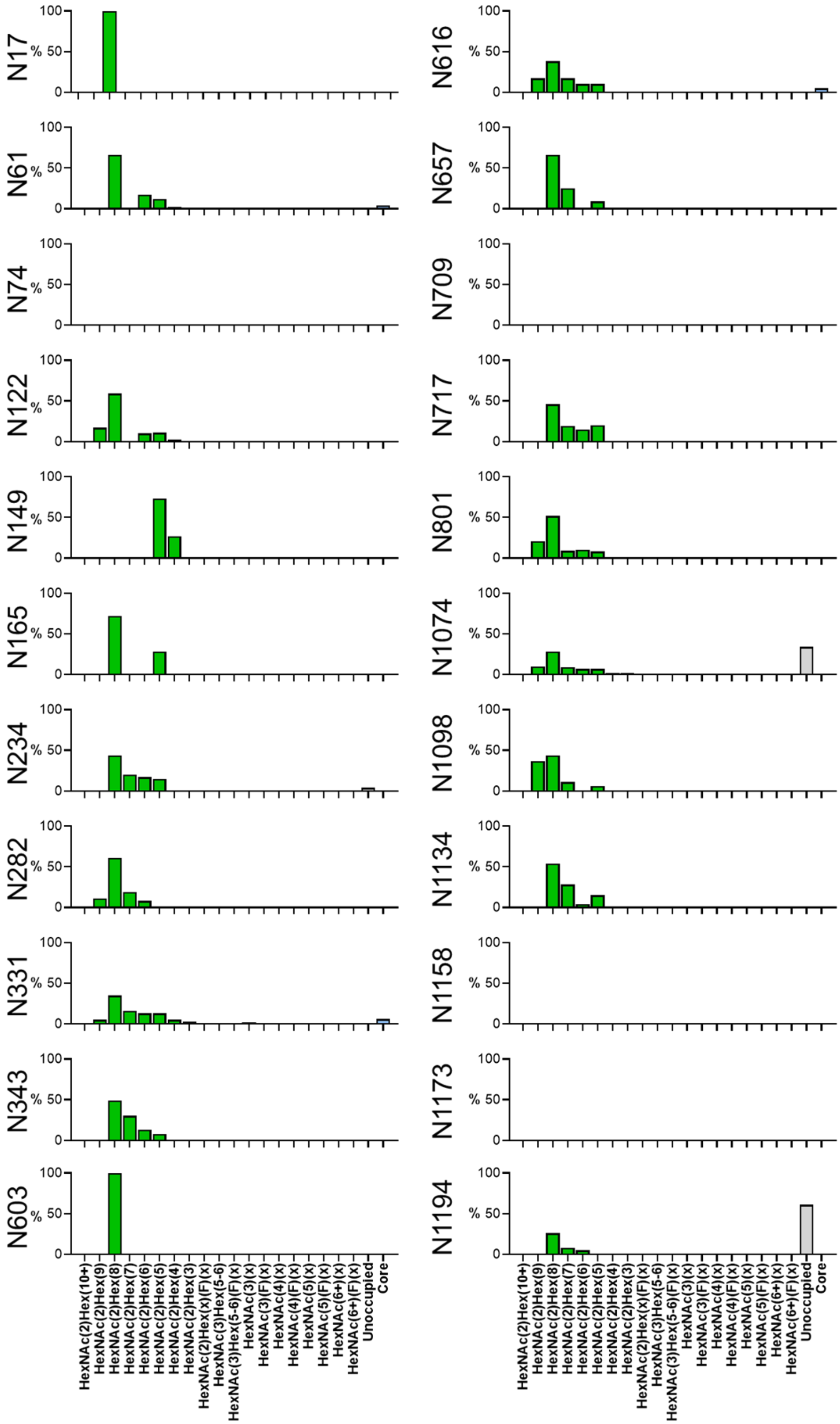
N17
N61
N74
N122
N149
N165
N234
N282
N331
N343
N603
N616
N657
N709
N717
N801
N1074
N1098
N1134
N1158
N1173
N1194
%
100
50
0
HexNAc(2)Hex(10+)
HexNAc(2)Hex(9)
HexNAc(2)Hex(8)
HexNAc(2)Hex(7)
HexNAc(2)Hex(6)
HexNAc(2)Hex(5)
HexNAc(2)Hex(4)
HexNAc(2)Hex(3)
HexNAc(2)Hex(x)(F)(x)
HexNAc(3)Hex(5-6)
HexNAc(3)Hex(5-6)(F)(x)
HexNAc(3)(x)
HexNAc(3)(F)(x)
HexNAc(4)(x)
HexNAc(4)(F)(x)
HexNAc(5)(x)
HexNAc(5)(F)(x)
HexNAc(6+)(x)
HexNAc(6+)(F)(x)
Unoccupied
Core

Supplementary Figure S2. Site-specific glycosylation of plant-produced spike. The data represents the composition of each N-glycosylation sequon as determined by LC-MS. The relative proportion of each glycoform is depicted as a percentage. Compositions containing Hex(10-12)HexNAc(2), glucosylated glycans, and Hex(9-5)HexNAc(2), composed of varying degrees of mannose processing, are classified as oligomannose-type N-glycans and are depicted in green. Hybrid-type glycans, those containing three HexNAcs and at least five hexoses, were colored as for complex-type glycans because one arm can be processed in a similar manner. Complex-type glycans were categorized according to the number of HexNAc residues detected and the presence or absence of fucose. Core glycans represent any detected composition smaller than HexNAc2Hex(3). The color codes in the schematic illustrates the processing state of glycans from least processed to most processed, oligomannose (green), hybrid (dashed pink), and complex glycans (pink). The proportion of unoccupied N-linked glycan sites are represented in grey.

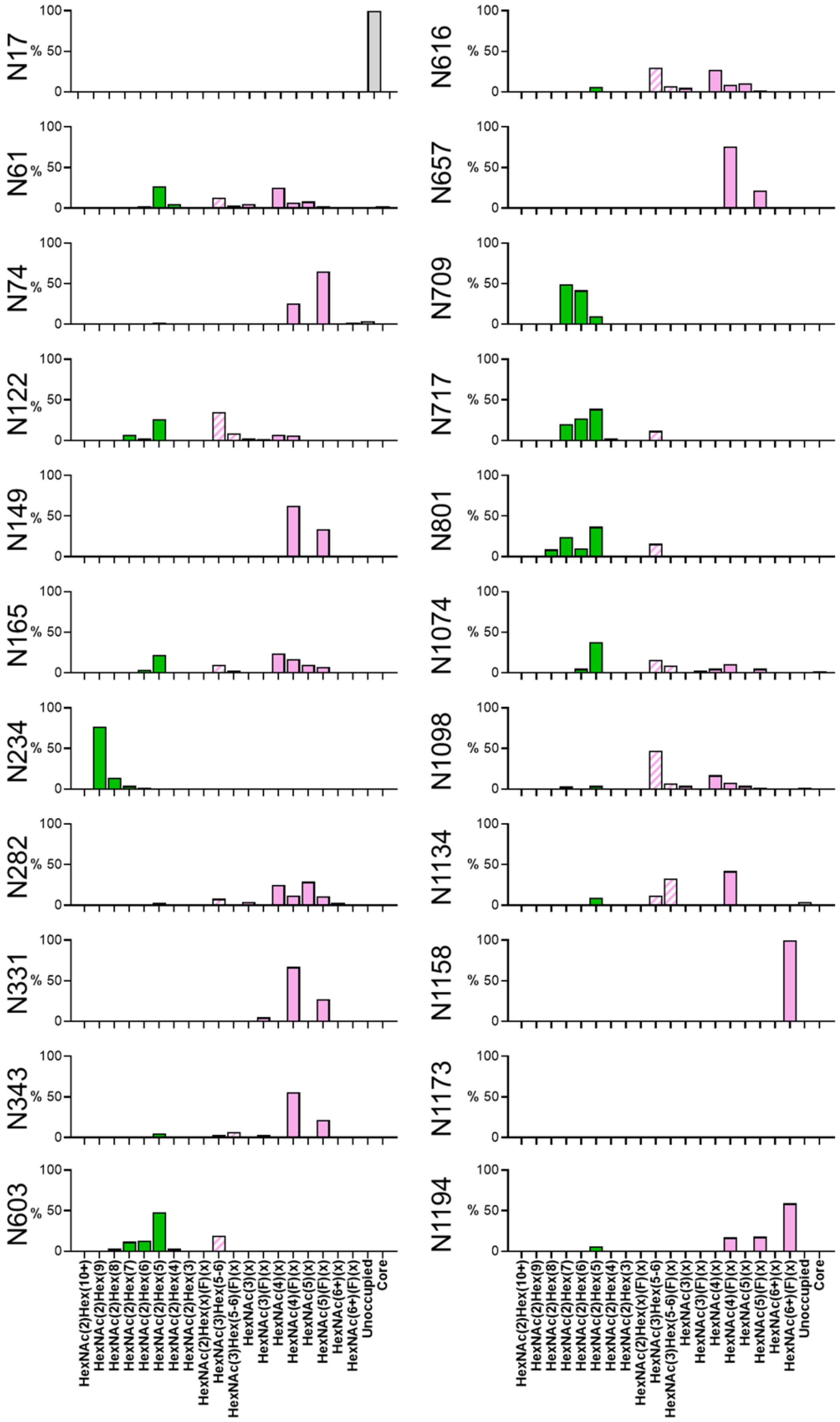
N17
N61
N74
N122
N149
N165
N234
N282
N331
N343
N603
N616
N657
N709
N717
N801
N1074
N1098
N1134
N1158
N1173
N1194
%
100
50
0
HexNAc(2)Hex(10+)
HexNAc(2)Hex(9)
HexNAc(2)Hex(8)
HexNAc(2)Hex(7)
HexNAc(2)Hex(6)
HexNAc(2)Hex(5)
HexNAc(2)Hex(4)
HexNAc(2)Hex(3)
HexNAc(2)Hex(x)(F)(x)
HexNAc(3)Hex(5-6)
HexNAc(3)Hex(5-6)(F)(x)
HexNAc(3)(x)
HexNAc(3)(F)(x)
HexNAc(4)(x)
HexNAc(4)(F)(x)
HexNAc(5)(x)
HexNAc(5)(F)(x)
HexNAc(6+)(x)
HexNAc(6+)(F)(x)
Unoccupied
Core

Supplementary Figure S3. Site-specific N-glycosylation of mammalian cell-produced spike. The data represents the composition of each N-glycosylation sequon as determined by LC-MS. The relative proportion of each glycoform is depicted as a percentage. Compositions containing Hex(10-12)HexNAc(2), glucosylated glycans, and Hex(9-5)HexNAc(2), composed of varying degrees of mannose processing, are classified as oligomannose-type N-glycans and are depicted in green. Hybrid-type glycans, those containing three HexNAcs and at least five hexoses, were colored as for complex-type glycans because one arm can be processed in a similar manner. Complex-type glycans were categorized according to the number of HexNAc residues detected and the presence or absence of fucose. Core glycans represent any detected composition smaller than HexNAc2Hex(3). The color codes in the schematic illustrates the processing state of glycans from least processed to most processed, oligomannose (green), hybrid (dashed pink), and complex glycans (pink). The proportion of unoccupied N-linked glycan sites are represented in grey.

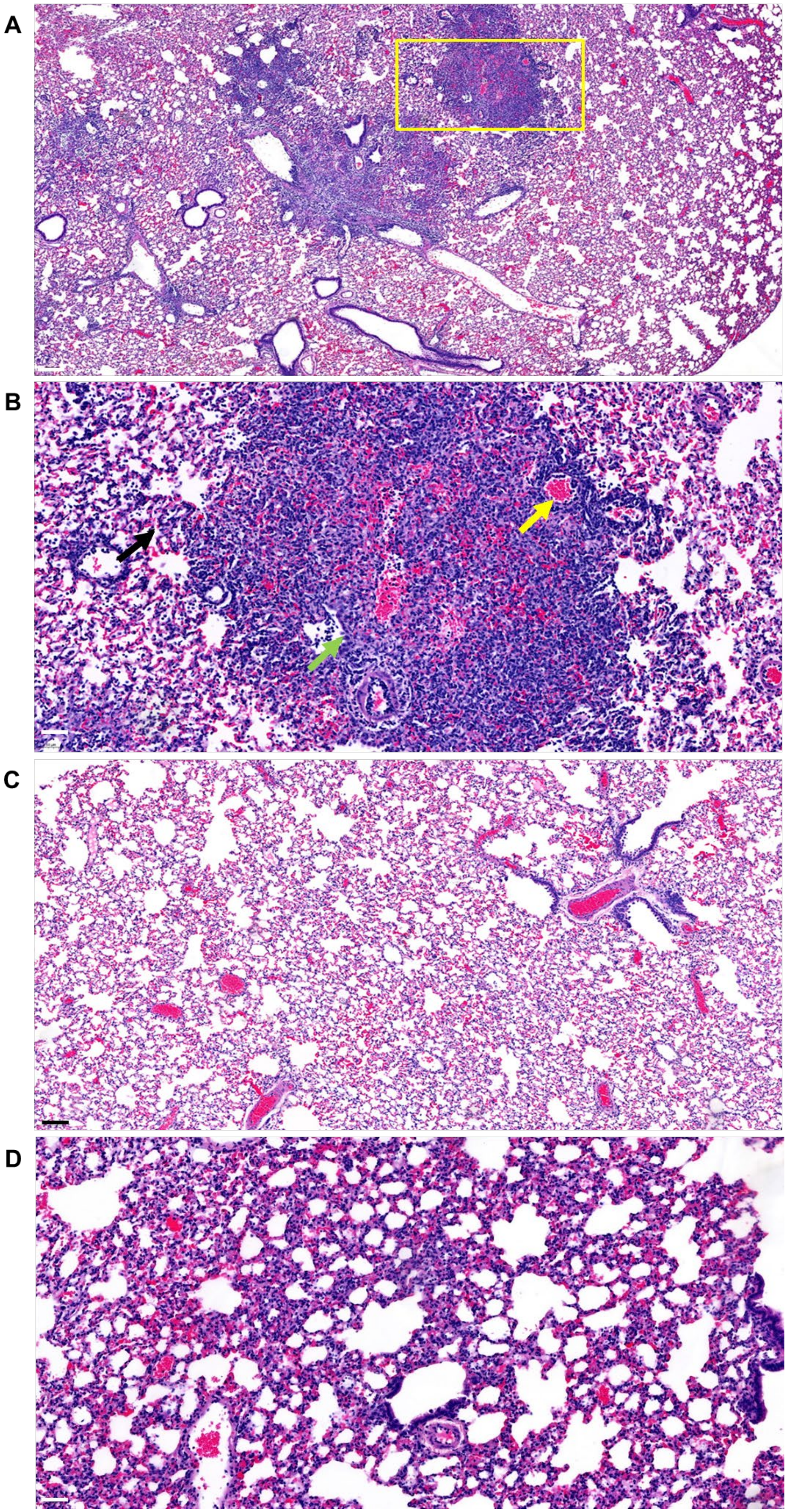
A
B
C
D

Supplementary Figure S4. Pathology following SARS-CoV-2 challenge of mock-vaccinated and vaccinated hamsters. A) H & E stained section of a lung of mock-vaccinated hamster (PBS control), showing marked lympho-plasmacytic infiltration and atelectasis centered around bronchiole (100x magnification). B) 200X magnification of region in the yellow rectangle from A. Green arrow = bronchiolitis; black arrow = alveolitis; yellow arrow = vasculitis/pervasculitis. C) H & E stained section of lung from hamster in Group 3 (vaccinated with mammalian spike protein) showing no abnormalities. No evidence of necrosis or inflammation (50X magnification). D) H & E stained section of lung from hamster in Group 2 (vaccinated with plant-derived spike protein) showing mild abnormalities (200x magnification). Scale bar = 50µm.

Supplementary Table S1. Site-specific N-glycosylation of recombinant spike produced in plants by integrated host and glyco-engineering.

	N17	N61	N74	N122	N149	N165	N234	N282	N331	N343	N603	N616	N657	N709	N717	N801	N1074	N1098	N1134	N1158	N1173	N1194
HexNAc(2)Hex(10+)	0	0		0	0	0	0	0	0	0	0	0	0		0	0	0	0	0			0
HexNAc(2)Hex(9)	0	0		17	0	0	0	11	5	0	0	17	0		0	21	10	37	0			0
HexNAc(2)Hex(8)	100	66		59	0	72	44	61	35	49	100	38	66		46	52	28	44	54			26
HexNAc(2)Hex(7)	0	0		0	0	0	20	19	16	30	0	17	25		19	9	9	11	28			8
HexNAc(2)Hex(6)	0	17		10	0	0	17	8	13	13	0	10	0		15	10	7	0	4			5
HexNAc(2)Hex(5)	0	12		11	73	28	15	0	13	8	0	10	9		20	8	7	6	15			0
HexNAc(2)Hex(4)	0	2		3	27	0	0	0	5	0	0	1	0		0	0	2	0	0			0
HexNAc(2)Hex(3)	0	0		0	0	0	0	0	3	0	0	1	0		0	0	2	0	0			0
HexNAc(2)Hex(x)(F)(x)	0	0		0	0	0	0	0	0	0	0	0	0		0	0	0	0	0			0
HexNAc(3)Hex(5-6)	0	0		0	0	0	0	0	1	0	0	0	0		0	0	0	0	0			0
HexNAc(3)Hex(5-6)(F)(x)	0	0		0	0	0	0	0	0	0	0	0	0		0	0	0	0	0			0
HexNAc(3)(x)	0	0		0	0	0	0	0	2	0	0	0	0		0	0	0	0	0			0
HexNAc(3)(F)(x)	0	0		0	0	0	0	0	1	0	0	0	0		0	0	0	0	0			0
HexNAc(4)(x)	0	0		0	0	0	0	0	0	0	0	0	0		0	0	0	0	0			0
HexNAc(4)(F)(x)	0	0		0	0	0	0	0	0	0	0	0	0		0	0	0	0	0			0
HexNAc(5)(x)	0	0		0	0	0	0	0	0	0	0	0	0		0	0	0	0	0			0
HexNAc(5)(F)(x)	0	0		0	0	0	0	0	0	0	0	0	0		0	0	0	0	0			0
HexNAc(6+)(x)	0	0		0	0	0	0	0	0	0	0	0	0		0	0	0	0	0			0
HexNAc(6+)(F)(x)	0	0		0	0	0	0	0	0	0	0	0	0		0	0	0	0	0			0
Unoccupied	0	0		0	0	0	4	0	0	0	0	0	0		0	0	34	1	0			61
Core	0	4		0	0	0	0	0	6	0	0	5	0		0	0	0	0	0			0
Oligomannose	100	96		100	100	100	96	100	90	100	100	95	100		100	100	66	99	100			39
Hybrid	0	0		0	0	0	0	0	1	0	0	0	0		0	0	0	0	0			0
Complex	0	0		0	0	0	0	0	4	0	0	0	0		0	0	0	0	0			0
Unoccupied	0	0		0	0	0	4	0	0	0	0	0	0		0	0	34	1	0			61
Fucose	0	0		0	0	0	0	0	1	0	0	0	0		0	0	0	0	0			0
NeuAc	0	0		0	0	0	0	0	0	0	0	0	0		0	0	0	0	0			0
Pentose	0	0		0	0	0	0	0	1	0	0	0	0		0	0	0	0	0			0

Supplementary Table S2: Site-specific N-glycosylation of recombinant spike produced in HEK293F cells.

	N17	N61	N74	N122	N149	N165	N234	N282	N331	N343	N603	N616	N657	N709	N717	N801	N1074	N1098	N1134	N1158	N1173	N1194
HexNAc(2)Hex(10+)		0	0	0	0	0	0	0	0	0	0	0	0	0	0	0	0	0	0	0		0
HexNAc(2)Hex(9)		0	0	0	0	0	77	0	0	0	0	0	0	0	0	0	0	0	0	0		0
HexNAc(2)Hex(8)		1	0	0	0	0	14	1	0	0	3	0	0	0	0	9	1	0	0	0		0
HexNAc(2)Hex(7)		1	0	7	0	0	4	1	0	0	12	0	0	49	20	24	1	3	0	0		0
HexNAc(2)Hex(6)		2	0	3	0	4	2	0	0	0	13	1	0	42	27	10	5	1	0	0		0
HexNAc(2)Hex(5)		27	2	26	1	22	1	3	1	5	48	6	1	10	39	37	38	4	9	0		6
HexNAc(2)Hex(4)		5	0	1	0	0	0	0	0	0	3	0	0	0	3	0	0	0	0	0		0
HexNAc(2)Hex(3)		1	0	0	0	0	0	0	0	0	0	0	0	0	0	0	0	0	0	0		0
HexNAc(2)Hex(x)(F)(x)		0	0	0	0	0	0	0	0	0	0	0	0	0	0	0	0	0	0	0		0
HexNAc(3)Hex(5-6)		13	0	35	0	10	0	8	0	3	19	30	0	0	12	16	16	47	12	0		0
HexNAc(3)Hex(5-6)(F)(x)		3	0	9	0	3	0	1	0	7	0	7	1	0	0	1	9	7	33	0		0
HexNAc(3)(x)		5	0	3	0	1	0	4	0	0	0	5	0	0	0	0	1	4	0	0		0
HexNAc(3)(F)(x)		1	0	2	1	0	0	0	5	3	0	0	1	0	0	0	3	1	0	0		0
HexNAc(4)(x)		25	0	7	0	24	0	25	0	1	0	27	0	0	0	1	5	17	0	0		0
HexNAc(4)(F)(x)		7	26	6	63	17	0	12	67	56	0	9	76	0	0	1	11	8	42	0		17
HexNAc(5)(x)		8	0	0	0	10	0	29	0	0	0	11	0	0	0	0	1	4	0	0		0
HexNAc(5)(F)(x)		2	65	1	34	7	0	11	27	22	0	2	22	0	0	0	5	2	0	0		18
HexNAc(6+)(x)		0	0	0	0	0	0	3	0	0	0	0	0	0	0	0	0	0	0	0		0
HexNAc(6+)(F)(x)		0	2	0	0	0	0	1	0	0	0	0	0	0	0	0	0	0	0	100		59
Unoccupied		0	4	0	0	0	0	0	0	0	0	0	0	0	0	0	1	2	4	0		0
Core		2	0	0	0	1	1	0	0	0	1	1	0	0	0	1	2	1	0	0		0
Oligomannose		36	2	37	1	27	99	6	1	7	80	7	1	100	88	80	46	9	9	0		6
Hybrid		15	0	44	0	13	0	10	0	10	19	37	1	0	12	17	26	54	45	0		0
Complex		47	94	19	98	59	0	84	99	83	0	55	99	0	0	2	25	35	42	100		94
Unoccupied		0	4	0	0	0	0	0	0	0	0	0	0	0	0	0	1	2	4	0		0
Fucose		12	94	18	99	27	0	25	99	90	0	18	99	0	0	2	28	18	75	100		94
NeuAc		12	48	23	60	24	0	14	45	18	0	7	56	0	2	8	8	58	22	75		69

Supplementary Table S3: Change in the site-specific N-glycosylation of recombinant spike produced in plants by integrated host and glyco-engineering compared to HEK293F.

GO vs HEK293 6P	N17	N61	N74	N122	N149	N165	N234	N282	N331	N343	N603	N616	N657	N709	N717	N801	N1074	N1098	N1134	N1158	N1173	N1194
HexNAc(2)Hex(10+)		0		0	0	0	0	0	0	0	0	0	0		0	0	0	0	0			0
HexNAc(2)Hex(9)		0		17	0	0	-77	11	5	0	0	17	0		0	20	10	37	0			0
HexNAc(2)Hex(8)		65		58	0	72	29	60	35	49	97	38	66		46	43	27	44	54			26
HexNAc(2)Hex(7)		-1		-7	0	0	16	19	16	30	-12	17	25		-1	-15	8	8	28			8
HexNAc(2)Hex(6)		15		7	0	-4	15	8	13	12	-13	10	0		-12	0	2	-1	4			5
HexNAc(2)Hex(5)		-15		-15	72	6	14	-3	12	3	-48	4	8		-18	-29	-31	2	6			-6
HexNAc(2)Hex(4)		-3		3	27	0	0	0	5	0	-3	1	0		-3	0	1	0	0			0
HexNAc(2)Hex(3)		-1		0	0	0	0	0	3	0	0	1	0		0	0	2	0	0			0
HexNAc(2)Hex(x)(F)(x)		0		0	0	0	0	0	0	0	0	0	0		0	0	0	0	0			0
HexNAc(3)Hex(5-6)		-13		-35	0	-10	0	-8	1	-3	-19	-30	0		-12	-16	-16	-47	-12			0
HexNAc(3)Hex(5-6)(F)(x)		-3		-9	0	-3	0	-1	0	-7	0	-7	-1		0	-1	-9	-7	-33			0
HexNAc(3)(x)		-5		-3	0	-1	0	-4	2	0	0	-5	0		0	0	-1	-4	0			0
HexNAc(3)(F)(x)		-1		-2	-1	0	0	0	-4	-3	0	0	-1		0	0	-3	-1	0			0
HexNAc(4)(x)		-25		-7	0	-24	0	-25	0	-1	0	-27	0		0	-1	-5	-17	0			0
HexNAc(4)(F)(x)		-7		-6	-63	-17	0	-12	-67	-56	0	-9	-76		0	-1	-11	-8	-42			-17
HexNAc(5)(x)		-8		0	0	-10	0	-29	0	0	0	-11	0		0	0	-1	-4	0			0
HexNAc(5)(F)(x)		-2		-1	-34	-7	0	-11	-27	-22	0	-2	-22		0	0	-5	-2	0			-18
HexNAc(6+)(x)		0		0	0	0	0	-3	0	0	0	0	0		0	0	0	0	0			0
HexNAc(6+)(F)(x)		0		0	0	0	0	-1	0	0	0	0	0		0	0	0	0	0			-59
Unoccupied		0		0	0	0	4	0	0	0	0	0	0		0	0	33	0	-4			61
Core		2		0	0	-1	-1	0	6	0	-1	4	0		0	-1	-2	-1	0			0

	N17	N61	N74	N122	N149	N165	N234	N282	N331	N343	N603	N616	N657	N709	N717	N801	N1074	N1098	N1134	N1158	N1173	N1194
Oligomannose		60		63	99	73	-2	94	89	93	20	88	99		12	20	20	90	91			33
Hybrid		-15		-44	0	-13	0	-10	1	-10	-19	-37	-1		-12	-17	-26	-54	-45			0
Complex		-47		-19	-98	-59	0	-84	-95	-83	0	-55	-99		0	-2	-25	-35	-42			-94
Unoccupied		0		0	0	0	4	0	0	0	0	0	0		0	0	33	0	-4			61
Fucose		-12		-18	-99	-27	0	-25	-97	-90	0	-18	-99		0	-2	-28	-18	-75			-94
NeuAc		-12		-23	-60	-24	0	-14	-45	-18	0	-7	-56		-2	-8	-8	-58	-22			-69
